# Treatment of fracture-related infection of the lower extremity with antibiotic-eluting ceramic bone substitutes: case series of 35 patients and literature review

**DOI:** 10.1007/s15010-020-01418-3

**Published:** 2020-04-08

**Authors:** Sebastian Pesch, Marc Hanschen, Frederik Greve, Michael Zyskowski, Fritz Seidl, Chlodwig Kirchhoff, Peter Biberthaler, Stefan Huber-Wagner

**Affiliations:** Department of Trauma Surgery, Klinikum Rechts Der Isar, School of Medicine, Technical University of Munich, Ismaninger Str. 22, 81675 Munich, Germany

**Keywords:** Fracture-related infection, Ceramic bone substitute, Antibiotic-eluting ceramic bone substitute, Bone infection, Cerament™ G

## Abstract

**Introduction:**

The current treatment concepts of fracture-related infection (FRI) [Consensus Conference (Anti-Infection Task Force (AITF)) on the definition of acute or chronic osteomyelitis (cOM)] are associated with unsolved challenges and problems, underlining the need for ongoing medical research.

**Method:**

Literature review of treatments for FRI and description of own cases.

**Results:**

We could include eight papers with 394 patients reporting treatments and outcome in FRI. The infection was resolved in 92.9% (mean) of all treatments. The mean follow-up was 25 months with a persistent non-union in 7% of the patients. We diagnosed 35 (19f/16m; 56.4 ± 18.6 years) patients with bone infections anatomically allocated to the proximal and distal femur (12×), the pelvis (2×), distal tibia (3×), tibial diaphysis (11×), the ankle joint (4×) and calcaneus (3×). These 35 patients were treated (1) with surgical debridement; (2) with antibiotic-eluting ceramic bone substitutes; (3) bone stabilization (including nail fixation, arthrodesis nails, plates, or external ring fixation), (4) optionally negative pressure wound therapy (NPWT) and (5) optionally soft tissue closure with local or free flaps. The mean follow-up time was 14.9 ± 10.6 months (min/max: 2/40 month). The overall recurrence rate is low (8.5%, 3/35). Prolonged wound secretion was observed in six cases (17.1%, 6/35). The overall number of surgeries was a median of 2.5.

**Conclusion:**

The results in the literature and in our case series are explicitly promising regarding the treatment of posttraumatic fracture-related infection.

## Introduction

Despite innovative treatment protocols developed during the last decades, posttraumatic fracture-related infection (FRI) is still associated with tremendous patient-related and socio-economic issues in the clinical setting [1–5].

Recently, Metsemakers and coworkers have coined the term ‘fracture-related infection’ (FRI) and introduced a consensus definition of posttraumatic bone infection. They defined several symptoms as confirmatory criteria of FRI (e.g., fistula, purulent drainage, positive culture of at least two separate deep samples) and stated other symptoms as suggestive criteria (e.g., clinical signs, radiological signs, single deep tissue specimen, elevated inflammatory serum markers, persistent or new wound drainage, new-onset of joint effusion) [6]. Their intention was to standardize the broad definition of acute and chronic osteomyelitis into one relatable list of criteria, which simplifies classification and the scientific comparability [4]. FRI is still a relevant problem, the rate of FRI is high (20–30% [7, 8]) with a reported treatment failure in 4–11% of patients [7].

Recently, promising results have been reported over the past years utilizing antibiotic-eluting ceramic bone substitutes for infection and dead space management with increasing success rates from up to 96% in osteomyelitis (Cierny–Mader class type I–IV) [9, 10].

In the past decade, a multi-stage protocol for FRI was used with multiple serial repetitive surgical debridements. Recently, McNally et al., postulated a single-stage protocol in the management of fracture-related infection [9]. This protocol demonstrates excellent clinical results with very low re-infection rates. By the use of an absorbable, antibiotic-eluting ceramic bone substitute, they effectively lowered the treatment time and the overall burden for the patients.

**Fig. 1:**
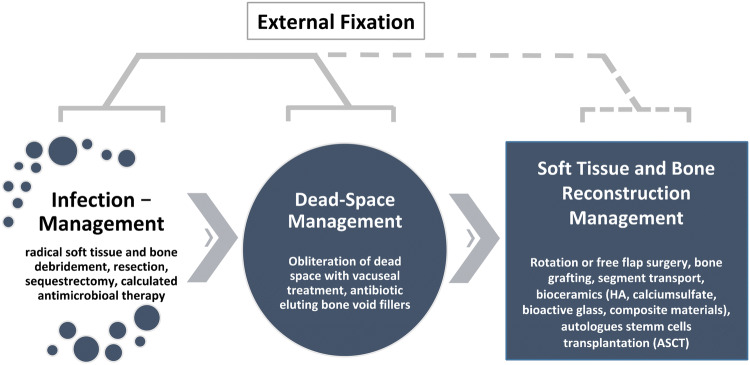
The treatment of FRI consists of consecutive management steps. The radical debridement of necrotic bone or devitalized sequestra is a key procedure for infection control accompanied by calculated antibiotic therapy. The implantation of a ceramic bone substitute with an antibiotic-eluting component is used for infection and dead space management. The osteoconductive feature of the ceramic bone substitute enhances the likelihood for bone healing and does not require surgical removal. Different methods for soft tissue and bone reconstruction are available with, e.g., bone segment transport (e.g., Ilizarov, hybrid fixation, PABST, Masquelet). During infection management, a rigid bone fixation is mandatory with temporal external fixation to ensure a supportive healing environment

In this manuscript, we reviewed the current literature for FRI based on the new consensus decision in chronic osteomyelitis. Furthermore, we present a case series of 35 patients with FRI treated in our department over the past 2.5 years. All patients were planned and treated according to our concept of management stages (see Fig. 1). The aim of this case series was to evaluate the usage of antibiotic-eluting ceramic bone substitute to potentially improve the success rate in septic bone surgery.

## Methods

### Review of the literature: search strategy

We conducted a condensed literature review of surgical treatments for patients with FRI for the period from 1990 through 2019. The search method in PubMed (https://www.ncbi.nlm.nih.gov/pubmed) was adopted as follows: fracture-related infection [Title] OR osteomyelitis [Title] AND antibiotic carrier [Title] OR antibiotic bone substitute [Title] OR drug-eluting bone substitute [Title] OR antibiotic ceramic [Title]. The search was performed within the title only in the new consensus definition of FRI and osteomyelitis with the application of local antibiotic-eluting carrier systems. A search for osteomyelitis in the complete text with all types of infection management protocols exceeds the purpose of this report. The data analysis focused on the patients’ characteristics (gender, age, risk factors and clinical history), clinical manifestation (microbiological/histological examination, anatomic allocation, fistula and purulent drainage) and therapeutic procedures (surgical protocol, re-infection, non-union, follow-up time and adverse effects). Two authors (M.Z., F.G.) independently screened 94 papers with the stated data analysis and published in the period from 1990 until 2019. The exclusion criteria were as follows: studies published in other languages than English, studies with no appropriate matching in the title or abstract, studies performed on carriers or substitutes without antibiotic characteristics, duplicated data or manuscripts without original data. Papers with insufficient statements about FRI in the title or abstract were separately screened through the entire text.

Regarding the former definition of osteomyelitis, hematogenous development of infection was not excluded in the review. Concerning the eligibility of study abstracts or titles, eight studies could be included with 394 patients treated for FRI with antibiotic-eluting carriers (see Table 1). The mean percentage of resolved infection, follow-up and non-union was calculated if applicable.

**Table 1:** Detailed summary of the literature review

Author	Patients	Mean age	Cierny–Mader classification	Investigated comorbidities	Diagnosis	Microbiological samples	Fistula/purulent drainage	Surgical protocol	Type of carrier	Re-infection	Follow up (month)	Non-union	Complication
Fiorenza et al. 2018 [22]	1	17	N/A	N/A	FRI femur	MRSA	Fistula	Single-stage	Porous alumina ceramic	0	14	None	None
McNally et al. 2016 [9]	100	51	CM III–IV	Class B hosts (CM)	FRI/ cOM extremity	MRSA, Staphylococcus	None	Single-stage	calcium sulphate with hydroxyapatite	4%	19	2%	Wound secretion, fracture
Kanakaris et al. 2014 [23]	24	44.5	CM1A–1B	N/A	cOM lower extremity	Staphylococcus	Fistula	Multiple stage	PMMA	4%	21	N/A	N/A
Ferguson et al. 2014 [12]	195	46	CM I–IV	Smoking	FRI/ cOM extremity	MRSA, Staphylococcus	None	Single-stage	Calcium sulphate	9.3%	44	N/A	Wound secretion, fracture
Franceschini et al. 2012 [24]	1	32	N/A	N/A	FRI tibia	Staphylococcus, Bacteroides	Fistula	Single-stage	Calcium sulphate/carbonate	0	12	None	None
McKee et al. 2010 [25]	30	45	CM I;III–IV	Smoking, Obesity, Diabetes mellitus, Osteoporosis, Drug abuse	FRI lower extremity	N/A	N/A	Multiple stage	Calcium sulphate/PMMA	14.3%	38	12.50%	N/A
McKee et al. 2002 [26]	25	43	CM I;III–IV	Smoking, diabetes mellitus, drug abuse	FRI lower/upper extremity	N/A	N/A	Multiple stage	Calcium sulphate	8%	28	8%	Wound secretion, fracture
Yamashita et al. 1997 [13]	18	38	N/A	N/A	FRI long bone	Staphylococcus, Streptococcus, Klebsiella	Fistula (9/18)	Multiple stage	Calcium hydroxyapatite	0	24–75	N/A	N/A
Mean	394							75.4% single-stage		7.14%	25	7.00%	
Pesch et al. 2020 new case series	35	56	CM I–IV	Diabetes mellitus, Smoking, Drug abuse	FRI lower extremity	Staphylococcus, Pseudomonas, *E. coli*	Fistula	Multiple/single-stage/muliple stage	Calcium sulphate with hydroxyapatite	8.5%	14	2.8%	wound secretion

### Case series

In this consecutive case series, we present 35 patients with posttraumatic FRI of the lower extremity attending our level I trauma center from March 2015 until June 2018. This study demonstrates results from a prospective, clinical trial in a single center study. The patients’ history, laboratory results, radiological reports, diagnostic tests, clinical symptoms, intraoperative biopsies and anti-microbial therapy were analyzed prospectively. The total number of surgeries, negative pressure wound therapy (NPWT) treatments and complications (prolonged wound secretion of antibiotic-eluting ceramic bone substitute, recurrence of infection) were collected. Bone consolidation during the follow-up was classified by a “modified radiographic union score (mRUS)”, which quantitatively evaluates the healing process and bone formation of four cortices in AP and lateral radiographic view (maximum bone healing 4 × 4 points) [11]. The nature of the study was a descriptive case series with no control group.

During infection and dead space management multiple repetitive intraoperative tissue biopsies were gathered, documented on a swap chart reflecting the anatomic landmarks. Data are provided as arithmetic mean and standard deviation. Statistical analysis was performed using the Sigma Stat 3.1 software (Systat Inc, Chicago, IL, USA).

All patients were individually planned for surgery according to our management concept, they all received antibiotic-eluting ceramic bone substitutes as combined dead space and reconstruction management.

The concept is based on a pre-operative interdisciplinary work-up with diagnostic imaging, the review of comorbidities and planning of the surgical approach. Our surgical management favors the single-stage protocol, as it is suitable for the individual circumstances and comorbidities of the respective patient. NPWT treatment was limited to a low number as needed. The overall surgical process is standardized by initial radical debridement of bone and soft tissue as necessary. The surgical interventions in our case series were performed by four different surgeons specialized in trauma surgery. Consecutively, temporary external/internal fixation to ensure best conditions for tissue recovery by rigid stabilization was performed. Despite primary wound closure, local free flaps were used for repair of soft tissue defects. The soft tissue restoration builds up a healthy biological environment.

The gentamicin-eluting ceramic bone substitute (e.g., Cerament™ G) facilitates dead space management. In detail, the gentamicin-eluting ceramic bone substitute was prepared according to the manufacturer’s guidelines and primarily cured for four minutes ex situ for best molding properties for dead space management. The pre-cured composite was then implanted inside the dead space, securing an excellent covering of the bone interface. The implantation requires a surface that is as dry as possible (e.g., facilitated by a tourniquet). The concentration level of the gentamicin-eluting ceramic bone substitute starts with an initial peak (> 1000 μg/mL) and is prolonged for at least 28 days (e.g., Cerament™ G, see https://www.bonesupport.com/en-eu/ceramentg/; 2019 April 10).

## Results

### Review of the literature

We could include eight papers reporting results in treatment of FRI in 394 patients (Table 1). The mean follow-up time was 25 months. The majority of those patients presented with 40–50 years of age. Six out of eight papers reported male and female cases. Two papers only presented male patients with single case reports. The paper with the largest study population could neither find significance with Cierny–Mader grade comorbidities nor smoking history in recurrent infection, but with polymicrobial infection [12]. The anatomic allocation of FRI was mostly in the lower extremity. In 295 patients, Staphylococcus aureus was cultured in 25.1–41.8% of the patients [9, 12]. Reported adverse effects included re-fracture of the treated bone in 3–4.6% of the cases [9, 12]. McNally and coworkers reported of six patients (6%) with prolonged white wound drainage after surgery caused by liquefied calcium sulfate [9].

In these 394 patients, the mean re-infection rate was 7.14%. In patients with infectious non-union, 93% consolidated by bone healing during the follow-up time of 25 months. A single-stage approach in FRI treatment was performed in 75.4% of all patients. This review is not described as following any agreed protocol and, therefore, not stated to be a comprehensive review, but it should provide a detailed overview about the most recent review from Ferguson et al. [10].

### Results of own case series

In our case series, we diagnosed 35 patients (19f/16m; 56.4 ± 18.6 years) with FRI anatomically allocated to the proximal and distal femur (12×), the pelvis (2×), distal tibia (3×), tibial diaphysis (11×), the ankle joint (4×) and calcaneus (3×).

These 35 patients were treated (1) with surgical debridement, (2) with Cerament™ G'; (3) bone stabilization (including nail osteosynthesis, arthrodesis nails, plates, or external ring fixation);’ (4) optionally VAC conditioning and (5) optionally soft tissue closure with local or free flaps.

The follow-up time was 14.9 ± 10.6 months (min/max: 2/40 months). The median follow-up time was 13 months. We observed very good clinical and radiological results by using gentamicin-eluting ceramic bone substitutes. The overall recurrence rate of infection was low (8.5%, 3/35). Delayed wound secretion (“white fluid”) was observed in six cases (17.1%, 6/35).

The median number of surgeries was 2.5. During multiple surgeries, several biopsies were taken.

According to the ASA risk classification, 13 patients were staged preoperatively ASA III, 15 ASA II and 7 patients ASA I. Furthermore, 13 patients were temporarily trans-fixated by an external fixation (e.g., Hexapod, Ilizarov ring fixator, unilateral modular pin to bar fixator). Specifically, 22 individuals primarily underwent implant removal due to assumed implant infection. In summary, 16 patients were treated by multiple NPWT treatments (mean of 1.2 ± 2.0 NPWT treatments). According to Cierny–Mader classification, the grade of osteomyelitis was subgrouped into grade I (4), II (7), III (14) and IV (10).

In detail, 19 patients (54.3%) received a one-stage infection management with implant removal and re-osteosynthesis with an intramedullary device or external fixation.

The mean period of hospitalization was 25.1 ± 17.6 days. In detail, the hospitalization time for one-stage management was 18.1 ± 14.0 days with a significant reduction in time (*p* = 0.0139). The grade of bone consolidation was evaluated routinely after 6, 12 and 24 weeks after surgery. The grading was grouped by the mRUS [11]. The full grade of consolidation (mRUS = 16) was seen in five individuals at all four cortices during follow-up. The other individuals showed different stages of consolidation with a mean mRUS of 8.5 score points of all 35 patients.

#### Microbiological results

The different biopsies mostly confirmed a staphylococcus infection (especially with Staphylococcus saprophyticus/pettenkofer/warneri/epidermidis and aureus) and evidence of Pseudomonas and Escheria coli.

Three patients were tested positive for 3-MRGN/MRSA infection. Furthermore, 34 patients received an additional intravenous (IV) antibiotic treatment during their hospital stay; one patient refused the prolonged antibiotic treatment. The patients initially and empirically received Meropenem in combination with Vancomycin IV based on the level of systemic concentration, which was routinely checked during their stay.

**Fig. 2:**
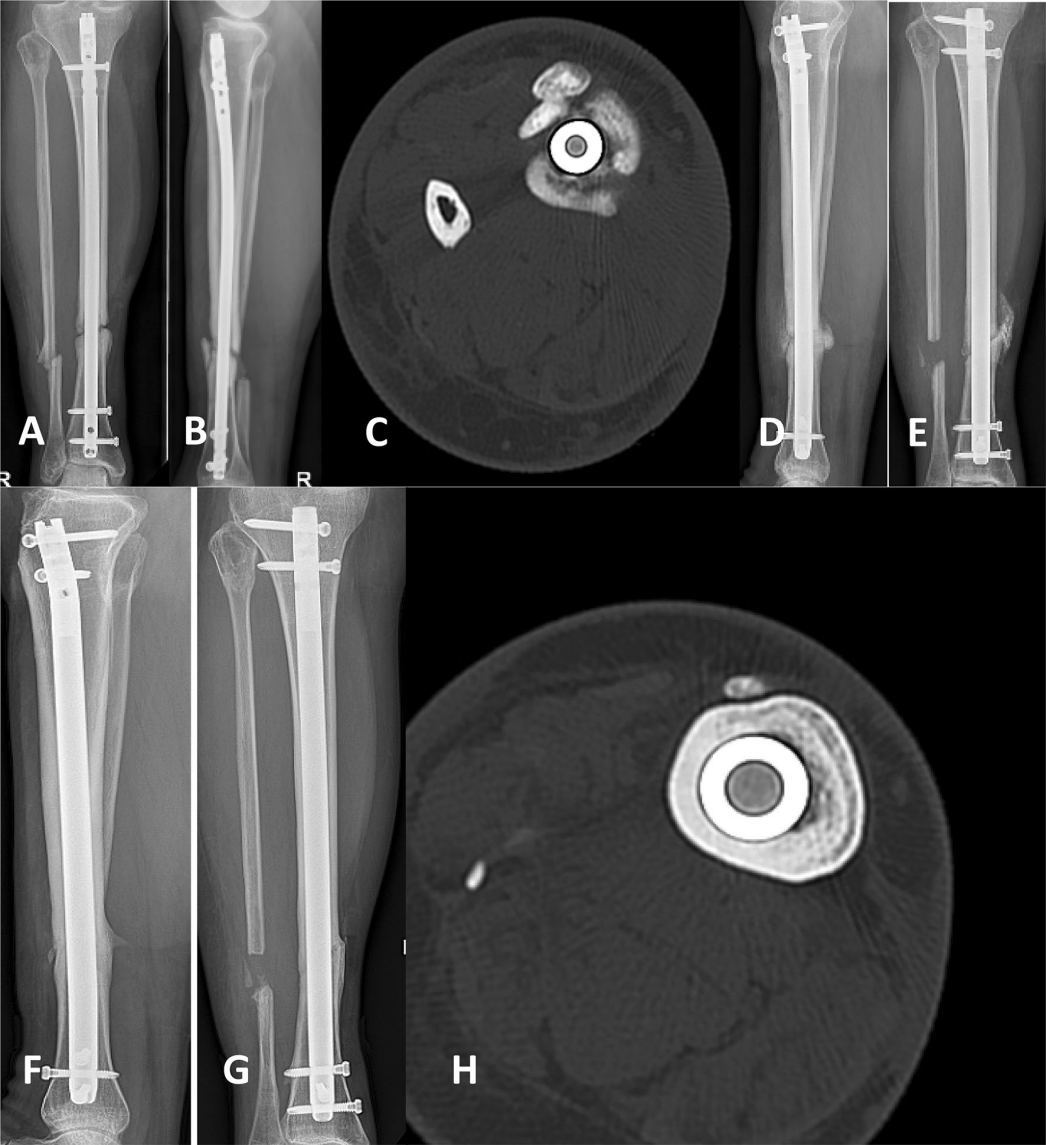
Late FRI 12 months after surgery with non-union of the tibial shaft after intramedullary nailing of a II° open tibial fracture (AO-42.A2). AP/lateral X-rays and axial CT scan showed non-union (**a**–**c**). After local debridement and intramedullary reaming, Cerament™ G was inserted, followed by internal stabilization with intramedullary nailing (**d**, **e**). After 18 months full bone consolidation was seen (**f**, **g**) as showed in AP/lateral X-rays and axial CT scan

**Fig. 3:**
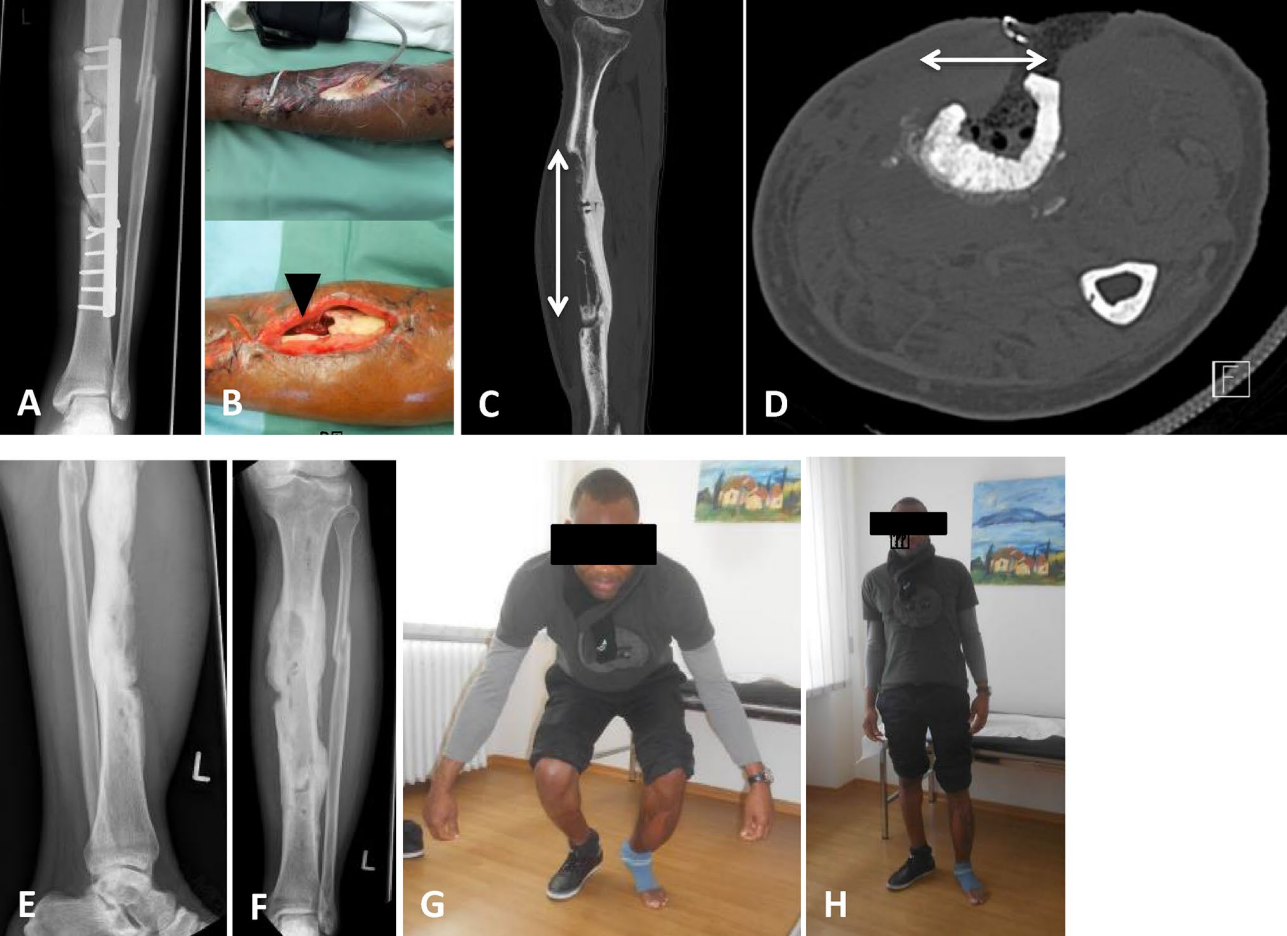
FRI after a heavy vehicle accident with a II° open tibial shaft fracture and initial fixation in Africa (**a**). Admission from foreign clinic after several debridements and NPWT treatments. A soft tissue (arrow mark in **b**) and bone (arrows in **c**, **d**) defect of around 18 cm was seen anterior to the tibial shaft. The tissue samples were positive for infection with *Staphylococcus epidermidis*, *Pseudomonas* and *Escheria coli*. After infection and dead space management (with Cerament™ G), the reconstruction management was completed with a local free flap (*M. latissimus* dorsi). Presentation 12 months after surgery with good bone consolidation and full weight-bearing (**e**–**h**)

**Fig. 4:**
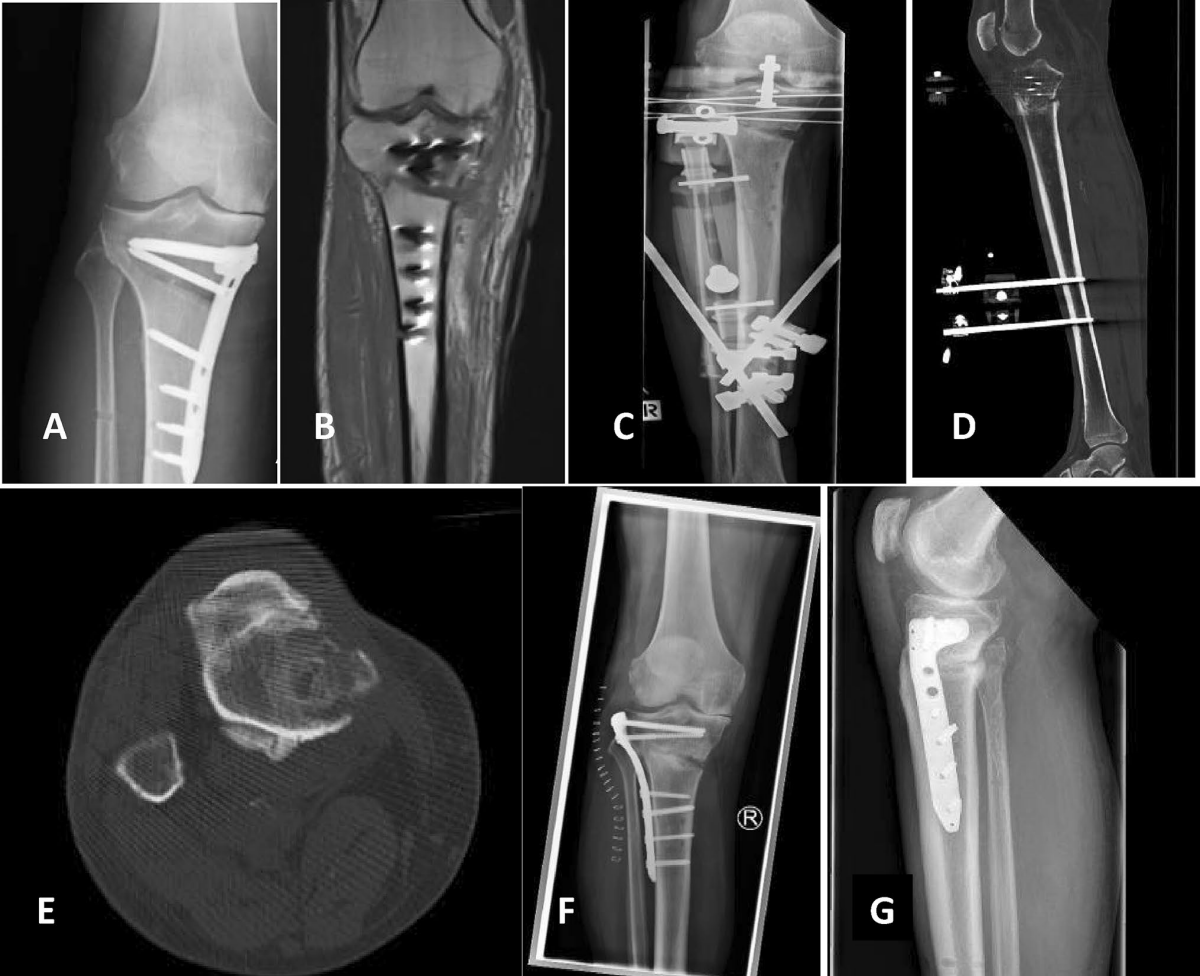
FRI after high tibial osteotomy (HTO) from a foreign clinic. Non-union of the osteotomy with multiple bony infarction at the proximal gap (**a**, **b**). Multiple bone samples were positive for Propionibacterium acnes. After debridement for infection management, Cerament™ G was inserted, followed by external hybrid fixation (**c**, **d**). Due to a good bone consolidation of the FRI (**e**), a conversion to plate fixation with a variable angle locking plate could be performed 5 months after external fixation (**f**, **g**)

**Fig. 5:**
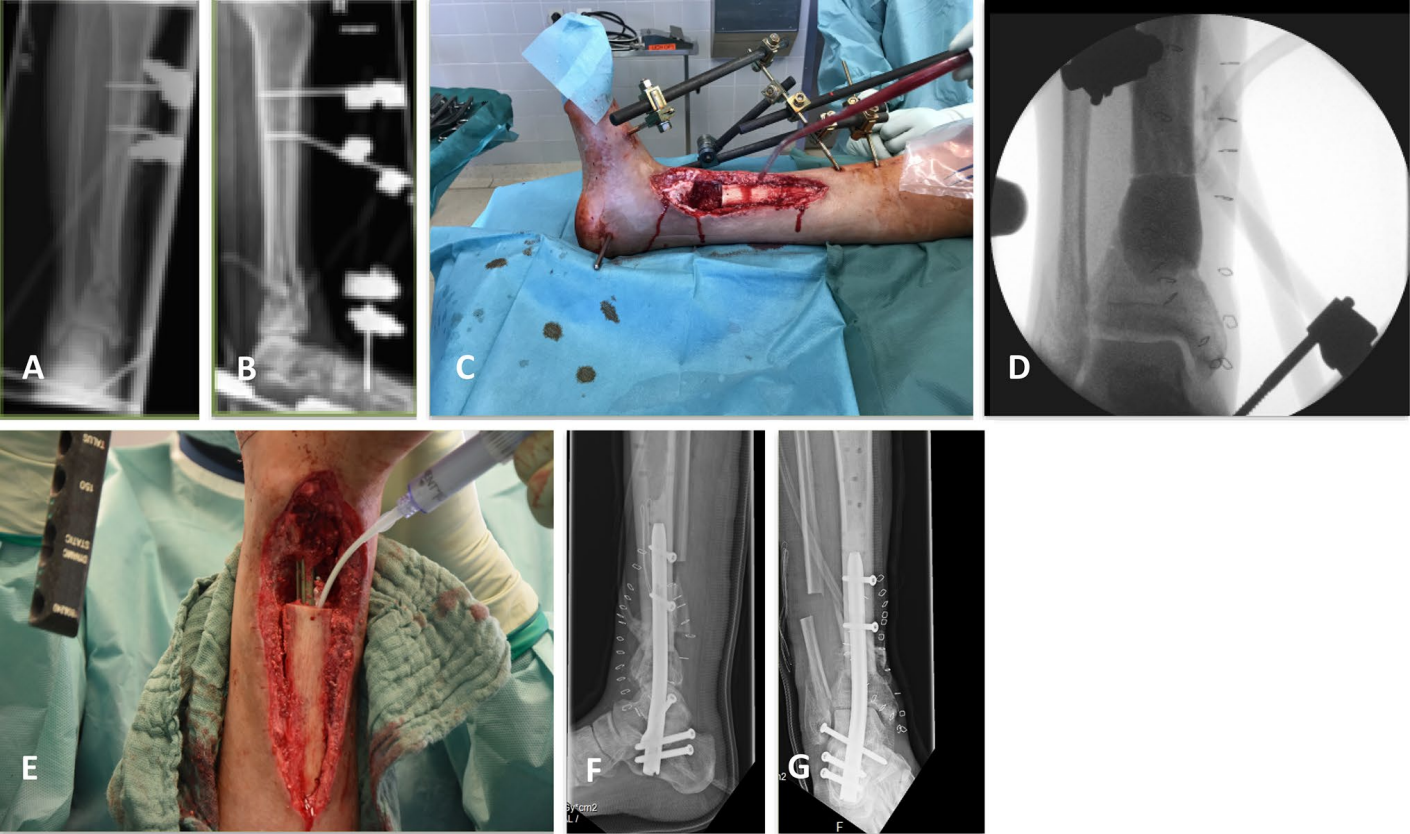
FRI after distal tibia fracture (AO.42-A2) admitted to our department with removed implants and external fixation with an anterior soft tissue defect at the tibia (**a**–**c**). Due to the broad infection management with debridement of 3 cm at the distal tibia, a Cerament™ G spacer was implanted (**d**). The swaps were positive for staphylococcus aureus and epidermidis. After eradication, an arthrodesis of the ankle was performed with retrograde nailing (**e**–**g**). Finally, a covering of the tibial soft tissue defect was achieved by a latissimus dorsi flap

Overall, we experienced only three cases (8.5%) of recurrent infection, which emphasizes the effectiveness of gentamicin-eluting ceramic bone substitute in our case series. A versatile compilation of successful FRI treatments with example cases are presented in Figs. 2, 3, 4 and 5.

#### Complications

Despite excellent treatments in FRI, a depiction of complications with recurrent infection in three individuals is provided in detail.

**Fig. 6:**
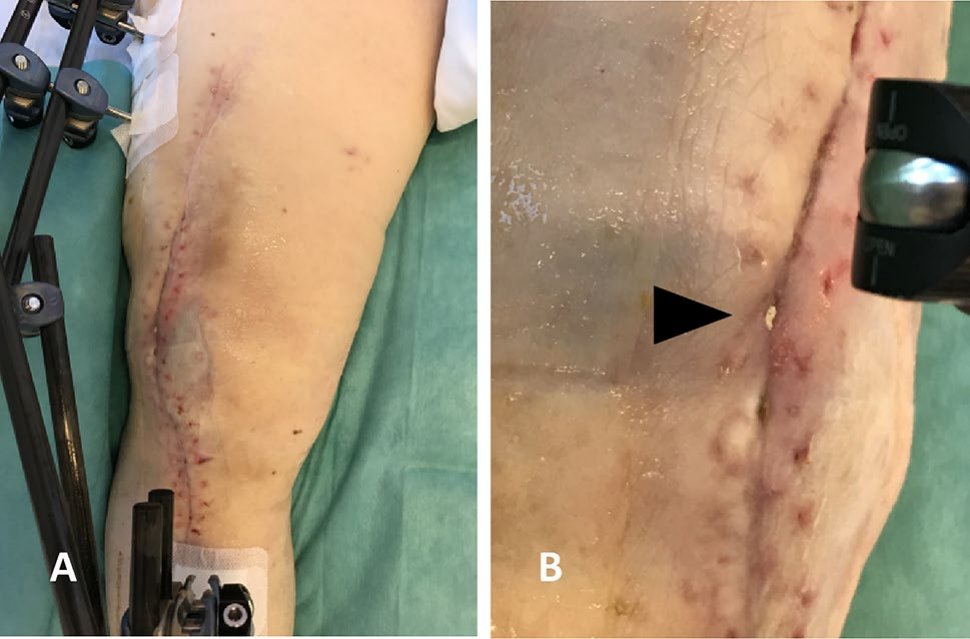
Prolonged local wound leakage of liquefied Cerament™ G in a FRI of the lower extremity (**a**, **b**)

One individual with infectious non-union at the femur after a gunshot wound showed a delayed wound healing at the distal femur. The patient initially received a large debridement of bone substance of the femur (4 cm). Dead space management was achieved by filling with gentamicin-eluting ceramic bone substitute and stabilization with internal plate fixation of the femur. Throughout, the patient showed increased “white fluid” secretion, which was suggestive of a degradation of the bone substitute and missing consolidation. A revision surgery was performed with implant removal (see Fig. 6).

Another patient with poly-drug abuse showed an early recurrence of infection after surgical therapy of an infectious non-union at the femur. Initially, the patient received a radical debridement of the infected bone with shortening of the limb and intramedullary retrograde nailing of the femur (leg lengthening/compression nail). During the hospital stay, the patient refused the additional IV antibiotic treatment. Four weeks postoperatively, the patient presented to our emergency department with early septic infection of the femur. Therefore, a complete implant removal and radical bone resection was necessary.

Our third case was a patient with late implant infection after a distal tibia fracture who was admitted to our department with FRI. Surgical debridement, implantation of a gentamicin-eluting ceramic bone substitute and external fixation were achieved. After 5 months, the patient was admitted with an infected pin tract in the proximal part of the tibia. A second debridement of infected bone in the distal tibia by infectious recurrence was performed, the initial Hexapod fixation of the distal tibia/ankle was removed and the patient was recommended to undergo callus distraction management. So far, no further complications were seen, and the docking site surgery was achieved 8 weeks later without complications.

**Fig. 7:**
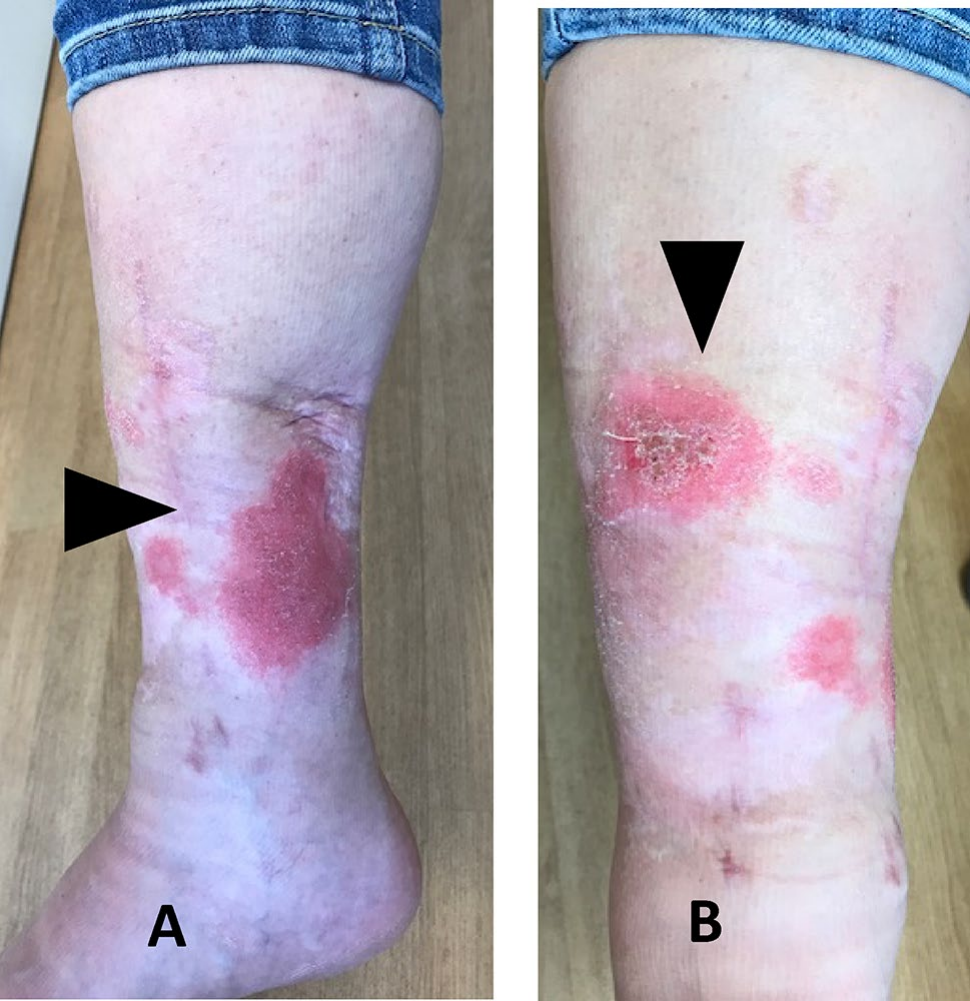
Local skin erythema (arrow mark) of the distal tibia maybe cause by an allergic reaction of intramedullary Cerament™ G in FRI of the lower extremity (**a**, **b**)

Besides these recurrences of infection, we could also observe one local allergic skin reaction (dermatologically confirmed), supposedly related to the gentamicin-eluting ceramic bone substitute in one patient with FRI of the tibia (see Fig. 7).

## Discussion

### Review of the literature

First, we performed a condensed review of the reported treatment concepts in the new consensus definition for FRI and second, we analyzed our own case series.

As an international expert group very recently proposed this definition, the former concept of osteomyelitis and osteitis was also included. The new definition for FRI provides surgeons with a clear and universal diagnostic tool to evaluate bone infections systematically. This systematical definition will introduce a standardization in diagnosis and treatment for FRI [6].

This study does not claim to be exhaustive for a systematic review. Rather, it compiles the results in treatment of FRI with antibiotic-eluting bone carriers over the past decades [10].

#### Evolution of treatment

The early reports of treatment in bone infection focused on multiple surgeries and non-degradable constructs of antibiotic-eluting carriers [i.e., Polymethylmethacrylat (PMMA)]. The proposition of calcium hydroxyapatites as a biodegradable antibiotic-eluting carrier rather than a cement “vehicle” impregnated with antibiotic, initially showed very excellent results in a single-center study by Yamashita et al. in the past century [13].

Nevertheless, the use of degradable bone substitutes as antibiotic carriers changed the treatment concept impressively, leading to a single-staged protocol with excellent results in resolving bone infection. The overall mean reinfection rate is low with 7.14% of the presented cases (27/378). The paradigm shift towards the application of degradable bone substitutes as antibiotic-eluting ceramic bone substitutes (i.e., degradable ceramics with gentamicin) facilitates the single-stage protocol, as no removal is needed after infection control. It needs to be emphasized that antibiotic-eluting ceramic bone substitutes are different regarding their product composition. We reviewed antibiotic-eluting ceramic bone substitutes consisting of calcium sulphate alone, calcium sulphate with hydroxyapatite and calcium hydroxyapatite. They all vary in their characteristics that will not be discussed in this review.

These management protocols demonstrate excellent results in the majority of the reported cases. This paper will not state the advantages of different ceramics in detail.

In general, FRI is a multifactorial problem that requires specific concepts of treatment to prevent secondary issues (20–30% persistent FRI [7]). In the literature, different concepts have been established including procedures of fixation, bone grafting and stabilization. Overall, there are many established and innovative bone defect management concepts following FRI (e.g., one-stage, two-stage, Masquelet, RIA, Ilizarov methods, different types of local antibiotic [8]).

#### New treatment concept

The complexity of this disease needs a comprehensive approach including thorough clinical assessment, radiological workup with CT, MRT, PET-CT for pre-operative planning and a well-elaborated individualized line of surgical treatments. Apart from the surgical therapy, which depends on the implant, the bone union and the general patient’s condition, the application of antibiotic treatment in FRI additionally supplements the chosen therapy. A prolonged antimicrobial therapy depends on the surgical procedure. After removal of an infected implant, an antimicrobial therapy is recommended for about 6 weeks, in retention of implant for 12 weeks [14]. Besides the infection management in FRI, antibiotic-eluting bone carriers also deliver a preventive activity in the infectious non-union treated in the single-stage management program. This concept should be well-discussed with the patient to rule out any informational misunderstandings.

#### Classification of FRI

In summary of the reported papers, the heterogeneity in different classification of FRI and various additional systemic antibiotic regimens after surgery cannot provide a standardized protocol for treatment of FRI and makes it difficult to compare the efficacy of the treatment. Recently, Metsemakers and colleagues published a new consensus definition for treatment of FRI based on an expert group decision [6]. In addressing this problem, a universal definition of FRI helps to improve the scientific comparability for treatment of FRI. Besides the definition of FRI, the current concepts of treatment are changing in quality and quantity, as the well-known multiple stage surgery for infection after fracture fixation switches towards a single-stage management with promising results. The usage of antibiotic-eluting ceramic bone substitutes instead of antibiotic PMMA spacers or debridement alone decreased the recurrence rate of infection markedly [9, 10].

### Case series

Second, we report on our own case series of patients with FRI and treatment with antibiotic-eluting bone substitute. To the best of our knowledge, this is the largest series within German-speaking countries. The majority of patients reviewed received an antibiotic treatment adopted to the cultural results for at least 6 weeks and up to 12 weeks [9, 12]. During the different case managements, we tried to limit the NPWT therapies to a minimum, whenever it was reasonable according to medical aspects.

We did not observe any increase of the reinfection rate for those patients that underwent surgery more than once. Of course, patients who underwent surgery in terms of a single-stage procedure had an advantage, at least regarding the hospital length of stay. Referring to socio-economic aspects, the reduced hospitalization time may also have an influence on the treatment costs [15]. The authors stated in their results that “deep infections” (focused on tibia fracture) increase the healthcare costs by 6.5-fold in infected patients. The overall infection rate after fracture fixation did not change over the past few decades, so they proposed a reduction of costs by minimizing, e.g. the length of hospital stay as one significant cost driver along with age, ASA score or delayed surgery [15, 16]. The mean length of hospital stay in our cases was 25.12 ± 17.6 days per patient compared to infected tibia fractures with a mean of 54 days. It should be emphasized that the comparison of cost factors differs in each healthcare system.

Literature demonstrates a decreasing number of surgical treatments per FRI per case in the past 30 years and the intention to limit these down to single-staged approaches (75.4% single stage). McNally et al. observed very good results with a single-stage infection management in FRI [9].

Regarding the single-stage approach, we were able to decrease the multiple debridements and NPWTs in our patients from the beginning and during this case series. We accept this as a kind of “learning curve” in our treatment of FRI, as we have continually improved our concepts.

In our case series, complications included prolonged wound secretion of “white fluid”, the degradation of calcium sulfate in six patients. It has to be emphasized that white fluid secretion is not equivalent to recurrent infection. It is neither equivalent to pus. This phenomenon was already noticed in previous studies [17, 18]. They reported the leakage as a liquefied calcium sulfate residue mainly in FRI of the tibia. We also observed this complication, mainly in FRI of the femur, tibia and calcaneus.

#### Bone healing

No re-fracture was noticed in our case series during the follow-up (0%). The re-fracture rate in the FRI cases of Ferguson was reported to be 4.6% [12]. In his cases, no further incidence of reinfection was noticed. However, we emphasize the recommendation of McNally et al. for cautious treatment with antibiotic-eluting bone substitute in large bone segment defects [9]. Nevertheless, no implant failure or secondary fracture dislocation could be observed in our case series. Clinically, the presence of weight-bearing without pain, full mobility and no local pain are also predictive for bone consolidation [19].

The “radiographic union scale in tibial fracture” (RUST) describes the consolidation of fractures in two radiographic views. Within this score, three consolidated cortices are used to describe full bone healing. A combination of radiological and clinical aspects should be evaluated in order to estimate the extent of bone consolidation [20].

The mean mRUS score of bone consolidation that was used in our case series was 8.5 score points. We evaluated four cortices in AP and lateral radiographic view. According to the score description, a value of 8.5 can be interpreted as a bone consolidation “in process” during the early bone remodeling phase (1–6 months). We emphasize that we report on a relatively short follow-up time of a mean of 14.9 months [11]. According to this radiological score, the full continuous and remodeled bone would result in a maximum of 16 (4 × 4) points. We observed five patients with a full bone consolidation (16 points mRUS score).

#### Additional antibiotic treatment

In comparison to the literature, 28.5% of our patients were culture positive for Staphylococcus aureus. There was no direct correlation of Staphylococcus aureus infection and recurring infection. According to the literature, 22–37.1% of microbiological samples showed no growth of bacteria; in our case series we could observe this result in 22.8% [12]. Regarding the clinical assessment, these patients also had either purulent sinuses or intraoperative purulence. One of the patients with recurrent infection refused the additional antibiotic treatment on the ward. A direct correlation of this neglect and recurrence of infection is not clear, but conceivable.

The systemic antibiotic therapy, which is given additionally to the local antibiotic agent, is mandatory in cases of fracture-related infections. The choice of the right antibiotic substance and the duration of therapy depends on the evaluation of the individual situation. Individual issues include the duration of previous infection, patient’s condition and type of pathogen and susceptibility. Thus, the general recommendation for the duration of systemic antibiotic therapy is 6–12 weeks after surgery [21].

The allergic complication with a skin reaction—supposedly due to Cerament™ G—might be triggered by a larger extra osseous leakage of calcium sulfate into the soft tissue around the FRI in the tibia. So far, no other allergic reactions were reported in the detailed review of the papers.

#### Study limitations

We realize that this study has limitations, as it is a retrospective analysis of cases. We accept that limitations, such as the low number of cases presented here cannot provide valid evidence for advantages or disadvantages, but it should emphasize the widespread availability and applicability of ceramic bone substitutes. Neither was a comparison with a control group made in our study hand. We also accept that the short follow-up with a mean time of 14.9 months limits the power of this retrospectively collected case series.

## Conclusion

Current literature demonstrates excellent results for the treatment of FRI by standard radical surgical debridement with additional local antibiotic treatment. Dead space and reconstruction management in these patients are well-studied and successful concepts are prevalent. In our case series, antibiotic-eluting ceramic bone substitutes promise a beneficial supplementary method for eradication of bacteria over surgical debridement. We emphasize the positive effect of the supportive treatment for FRI. However, insights into the long-term outcome following this innovative treatment concept are lacking; nevertheless, the excellent short-term follow-up results are promising.
